# Giant osteoclasts in patients under bisphosphonates

**DOI:** 10.1186/1472-6890-14-31

**Published:** 2014-07-08

**Authors:** Fabrice Mac-Way, Andrea Trombetti, Christian Noel, Marie-Hélène Lafage-Proust

**Affiliations:** 1 Laboratoire de Biologie Intégrative du Tissu Osseux, Inserm U1059, Université de Lyon, Saint-Etienne, France; 2 Division of Nephrology, CHU de Québec, L’Hôtel-Dieu de Québec Hospital and Research Center, Faculty of Medicine, Laval University, Quebec, Canada; 3 Department of Internal Medicine, Centre Hospitalier Universitaire de Genève, Geneva University, Geneva, Switzerland; 4 Renal Division, CHU-Hôpital Calmette, Lille University, Lille, France

**Keywords:** Bisphosphonate, Giant osteoclast, Bone biopsy, Bone resorption, Osteoporosis

## Abstract

**Background:**

Bisphosphonates have been widely used for treatment of high bone resorption states. It lowers bone turnover by inhibiting osteoclasts bone resorption with various mechanisms of actions: inhibition of osteoclast formation and attachment to the bone surface, induction of metabolic injury, alteration of vesicle trafficking and induction of osteoclast apoptosis. Bone biopsies studies from patients under bisphosphonates have shown that some resorption parameters are decreased as expected but the number of osteoclasts seems not to be necessarily decreased. The description of osteoclasts morphology from patients treated with bisphosphonates has rarely been reported in the literature.

**Case presentation:**

We describe in this paper two patients treated with bisphosphonates from whom iliac crest bone biopsies have shown large, multinucleated and apoptotic osteoclasts that were not associated with bone resorption activities. The characteristics of these osteoclasts are described and the literature reviewed.

**Conclusion:**

The appropriate recognition of these giant osteoclasts in bone tissues from patients treated with bisphosphonates is of primary importance for bone pathologists and should not be interpreted as signs of increased bone resorption as seen in hyperparathyroidism, bone cancer or Paget’s disease of bone.

## Background

Bisphosphonates (Bps) constitute a major class of drugs used to treat various metabolic bone diseases including osteoporosis, metastatic or post-transplant bone disease. Since their introduction more than thirty years ago, many studies have shown that they mainly act through inhibition of osteoclasts (OC) bone resorption: inhibition of OC formation [1] or attachment to the bone surface [2], induction of metabolic injury to OC [3], alteration of vesicle trafficking and/or induction of OC apoptosis [4]. The anti-osteoclastic effects of nitrogen-containing Bps (including alendronate (ALN) and risedronate (RIS)) result from inhibition of the enzyme farnesyl pyrophosphate synthase that ultimately leads to OC death [5]. The decrease in circulating markers of bone resorption in Bps-treated patients has been extensively documented, using measurements of collagen breakdown products in urine or serum [6] and has been used to monitor patients compliance or predict Bps efficacy [7]. While bone histomorphometry studies mentioned the reduction of resorption rate [8], period [9] or lacunae depth [10] in patients treated with Bps, the number of OC has been reported to be lowered, normal or even increased. In fact, the description of their morphology has rarely been reported in the literature. In this paper, we report two cases in which bone biopsies from patients under Bps showed unusual giant, multinucleated OC that were not associated with resorption activity. These OC are characterized and current literature is reviewed.

## Case presentation

### Case 1

**Figure 1:**
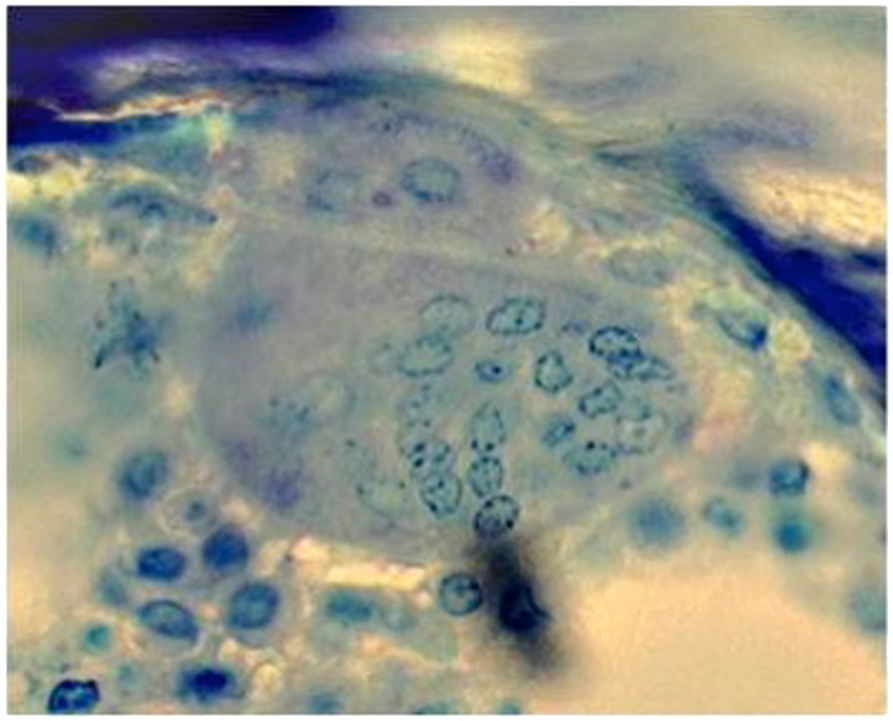
**Giant OC in patient 1 from TRACP staining (400X magnification).** Note the absence of bone resorption, multinucleated and detached OC from bone surface.

A 41 year-old woman known for a relapsing Cushing syndrome with two previous unsuccessful pituitary surgeries and multiple fractures history underwent an iliac crest bone biopsy while under oral Bps for three years. Six and four years before, she suffered respectively from bilateral metatarsal fractures and a spontaneous right femoral neck fracture. After right hip surgery, oral calcium (500 mg twice daily), vitamin D (400 i.u twice daily) and ALN (70 mg/week) were started (see Table 1 for period under treatment). Subsequently, she suffered from two other episodes of fractures: left ischium and left femoral neck fractures. Each of her hospitalisations included biochemistry tests that were normal except for an elevated blood cortisol (>744 nmol/l, normal range: 120-620 nmol/l) and osteopenia (Table 1) so she finally underwent a successful total hypophysectomy in the year preceding the bone biopsy. Because she continued to fracture while under Bps, she was switched from ALN to teriparatide (TPTD) soon after her bone biopsy. Bone mineral density (BMD) measurement 6 months and two years after TPTD introduction showed normal T-score values but unfortunately she continued to experience fractures while under TPTD: left foot (two times) and right ischium. After 18 months of TPTD treatment, she was put back on Bps (RIS 35 mg/week) for the following two years. Still, she suffered from right metatarsal and left cuneiform fractures so RIS was furtherly changed for ranelate strontium. The histomorphometric parameters are reported in Table 2. The Bone Formation Rate (BFR) was decreased as expected under Bps but OC number, as assessed by counting TRACP (Tartrate Resistant Acid Phosphatase) positive cells attached to the bone surface, was not diminished. Up to three giant OC with more than 25 nuclei/cell were observed per slide and most of them were detached from the bone surface [Figure 1]. Osteoid bone and marrow cavity were normal and no woven bone was observed throughout the biopsy ruling out hyperparathyroidism or Paget’s disease of bone.

**Table 1:** Evolution of biological parameters and BMD in patient 1

	**-48 months**	**-24 months**	**-12 months**	**Bone biopsy**	**-6 months**	**-12 months**	**-36 months**
Deoxypyridinoline (8.2-20.2 nmol/mmol creat)	16.8	4.3	8.3	8.9	24.1	14.6	6.9
Intact PTH (10-65 pg/mL)	27.4	ND	ND	37.3	ND	52.1	65.1
25-(OH) D (20-90 nmol/L)	45	ND	ND	57	ND	79	94
Alkaline phosphatase (30-125 U/L)	72	ND	ND	53	97	93	47
BMD (T-score)							
Lumbar	-1.2	-1.3	ND	-1.1	-1.2	-0.8	ND
Femoral neck	-0.6	-0.7		-0.4	-0.2	-0.6	

PTH: Parathyroid hormone; BMD: Bone mineral density; ND: not determined.

**Table 2:** Histomorphometric parameters for patient 1 and 2

**Parameters**	**Patient 1**	**Patient 2**	**Normal range**
BV/TV (%)	37.3	22.3	21.9 ± 3.3
OV/BV (%)	0.6	0.6	2.0 ± 1.2
MAR (μm/j)	0.92	0.53	0.72 ± 0.12
MS/BS (%)	2.9	5.9	7.0 ± 4.1
MLT (j)	19.7	23.6	24.5 ± 7.5
BFR (μm3/μm^2^/j)	0.0267	0.0310	0.0435 ± 0.060
OcN/BA (cell/mm^2^)	3.44	8.91	3.80 ± 1.70
Oc S/BS (%)	3.1	2.8	1.4 ± 0.5

BV/TV: Bone volume/Tissue volume, OV/BV: Osteoid volume/Bone volume, MAR: Mineral Apposition Rate, MS/BS: Mineralizing surface/Bone surface, MLT: Mineralization Lag Time, BFR: Bone Formation Rate, OcN/BA: Osteoclasts number/Bone area, OcS/BS: Osteoclasts surface/Bone surface.

Note the absence of mineralization defect (normal MLT) in both patients and normal or increased OC number.

### Case 2

**Figure 2:**
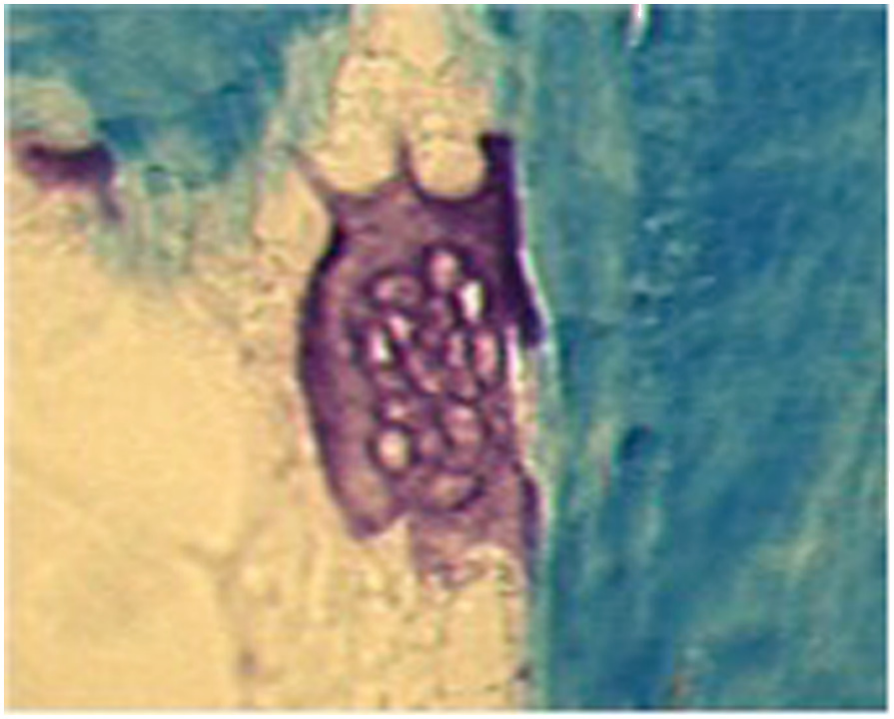
**Giant OC in patient 2 from TRACP staining (200X magnification).** Note that these giant OC have the same characteristics as seen in patient 1.

The second patient was a 41 year-old woman with renal insufficiency due to an obstructive nephropathy. She started hemodialysis at age 24 and underwent a successful renal transplantation at age 38. When she was on hemodialysis, her BMD score was already reduced that was confirmed 6 months after transplantation with elevated parathyroid hormone (PTH) and reduced 25-(OH)D (Table 3, - 36 months). Oral vitamin D (400 i.u twice daily) was started soon after transplantation and RIS (35 mg/week) was given one year after when renal function was stable. During follow-up, as the BMD was still low after normalization of biological parameters (PTH and 25-(OH)D), an iliac crest bone biopsy was performed while she was under RIS for 26 months. The bone histomorphometry results showed a reduced BFR in agreement with Bps treatment [Table 2] while OC number was slightly increased. Again, multinucleated giant OC were observed, most of them being detached from the bone surface [Figure 2]. They were not associated with resorption activity and were absolutely identical to those seen in patient 1. Hyperparathyroidism bone disease and Paget’s disease of bone were again ruled-out since not abnormalities were reported for osteoid and marrow cavity.

**Table 3:** Evolution of biological parameters and BMD in patient 2

	**-36 months**	**-24 months**	**-12 months**	**Bone biopsy**	**+3 months**
CTX (250–4500 pmol/L)	13700	ND	4760	3650	6545
Intact PTH (10–65 pg/mL)	127	ND	111	51	59
25-(OH) D (20–90 nmol/L)	22	40	60	62	82
Bone Alkaline					
Phosphatase (11.6-30.6 U/L)	21	7	9	10	11
BMD (T-score)					
Lumbar	-2.6	ND	ND	-3.3	ND
Femoral neck	-3.1			-3.5	

CTX: Carboxy-terminal collagen crosslinks; PTH: Parathyroid hormone; BMD: Bone mineral density, ND: not determined.

## Discussion

In this report, we described large multinucleated OC on the edge of bone trabeculae observed on bone biopsies from two patients under Bps therapy. These patients were treated with ALN and RIS for respectively 38 and 26 months. These OC had an average length of 92 μm, mostly detached from bone surface and were not associated with resorption lacunae. No mineralization defects were seen in both patients (normal osteoid volume and mineralization lag time) and OC number was either normal or increased. We observed 2 or 3 of these giant OC per slide that are not usually accounted for OcN/BA determination. Furthermore, these large OC had an apoptotic appearance with a clear nuclear fragmentation and nuclear peripheral beading when stained with DAPI (4′-6-Diamidino-2-phenylindole, data not shown). The BFR was decreased as expected in both patients as well as the biochemical markers of bone resorption while under Bps. The osteoid bone, bone marrow cavity and collagen structure were all well characterized for appropriate diagnosis to rule out other bone pathologies such as hyperparathyroidism, Paget’s disease of bone or neoplasia.

The first paper describing similar OC morphology was published in the New England Journal of Medicine in which the authors reported these giant OC in patients treated with ALN [11]. They reported the results of a 3-year prevention trial of ALN in postmenopausal women [12]. Fifty-one bone biopsies were performed and they described that 27% of OC seen in these biopsies were giant, multinucleated and detached from bone surface with pyknotic nuclei. They found an increased OC number that correlated with the cumulative ALN dose and concluded that long-term ALN treatment was associated with increased OC number but some of them were undergoing apoptosis.

To better describe the phenotype of these OC, a review was published in which the authors explain the characteristics of these giant OC seen from patients under Bps [13]. They reported the presence of these giant OC from iliac crest bone specimen of a 55 year-old woman treated with an aminobisphosphonate for 4 years and they discuss the differential diagnosis including Paget’s disease of bone, hyperparathyroidism, giant cell tumor and fibrous dysplasia. They specify the unique and common characteristics of these OC morphology: often more than 40 nuclear profiles compared to 2–8 nuclear profiles found in normal OC, detachment from the bone surface and up to 30% apoptotic.

Recently, Jobke et al have reported the same description as ours in osteoporotic patients treated with nitrogen-containing Bps during 3 years [14]. They also described a normal number of OC in these patients that is consistent with our histomorphometry results. These giant OC were exactly similar to our description and the one provided previously.

By our description and those made by others, it seems therefore plausible that the presence of these non-resorbing giant OC are directly related to the mechanism of action of the Bps. It has already been shown that nitrogen-containing Bps interfere with farnesyl pyrophosphate synthase in the mevalonate pathway resulting in a reduced geranylgeranyl diphosphate that is required for the prenylation of Guanosine triphosphate binding proteins, hence preventing formation of the ruffled border of the OC [15,16]. This leads to cytoskeletal malfunction and ultimately death by apoptosis. The decrease in OC resorption capability under Bps therapy could delay OC apoptosis allowing the fusion of OC precursors and produce these large multinucleated and apoptotic OC as proposed by Weinstein et al [11]. In this view, it is therefore not surprising that OC number was reported to be normal or even increased in our patients. Indeed, this was already noted in animal studies where RIS and ALN inhibited bone resorption at doses tenfold lower than what were required to reduce OC number [16]. In ALN treated rats, multinucleated OC that exhibited poorly organized ruffled borders and detached from the bone surface have also been reported [17]. It is important to note that these giant OC are seen not only in patients treated with nitrogen-containing Bps but also with amino-Bps as in our case. The identification of giant multinucleated OC in bone specimens from patients treated with Bps is of primary importance as it should not be interpreted as markers of increased bone resorption. Indeed, the absence of resorption capability, the detachment from bone surface and the presence of apoptosis markers characterise these OC and their presence should not be identified as active OC. Although our patients were different from the cases described in the literature since they had secondary cause of osteoporosis, our findings are nevertheless identical to previously reported cases.

## Conclusion

Bisphosphonates decrease bone resorption by inducing OC apoptosis and often allow appearance of multinucleated and giant OC that can be observed on patients bone tissue. The recent description of these OC gives us a better understanding on their mechanisms of action and is essential to recognise for all bone pathologists. Indeed, these giant OC should not be interpreted as markers of increased bone remodelling, which could lead to a false diagnosis of Paget’s disease of bone, hyperparathyroidism bone disease or even bone tumour.

## Consent

Written informed consent was obtained from the two patients for publication of this Case report and any accompanying images. A copy of the written consent is available for review by the Editor of this journal.

## Competing interests

The authors declare that they have no competing interests.

## Authors’ contributions

Data collection: FM, AT, CN, MLP. Data interpretation: FM, MLP. Drafting and revising manuscript: FM, AT, CN, MLP. Final approval: FM, AT, CN, MLP. All authors have read and approved the final manuscript.

## Pre-publication history

The pre-publication history for this paper can be accessed here:

http://www.biomedcentral.com/1472-6890/14/31/prepub
